# Patient dissatisfaction associated with physician-patient linguistic discordance in California clinics: an analytical cross-sectional study

**DOI:** 10.1186/s12913-023-09176-2

**Published:** 2023-02-23

**Authors:** Miguel A. Fernández-Ortega, Arturo Juárez-Flores, Gustavo A. Olaiz-Fernández, Daniel A. Muñiz-Salinas, AB1045 Law Analysis Group, Mario E. Rojas-Russell, Efrén R. Ponce-Rosas, Félix J. Vicuña-de-Anda, Arturo Aguirre-Gamero, Armando Manzanilla-Romero, Juan C. León-Rodríguez, Elena Gómez-Peña, Maximiliano Cuevas, Gagan Pawar, Rosa V. Fernández, Graciela Soto, Yamili Espejo-Iriarte, Omar Rodríguez-Mendoza

**Affiliations:** Centro de Investigación en Políticas, Población y Salud, Facultad de Medicina, Universidad Nacional Autónoma de México, Ciudad de México, Mexico

**Keywords:** Patient satisfaction, Primary health care, Communication barriers, Emigration and immigration, Health services accessibility, Language

## Abstract

**Background:**

Patient satisfaction is considered as a product of two psychological processes, a cognitive one, including expectations and perceptions, and an emotional one resulting from the congruence between expectation and subjective perception of the user. The objective was to identify the factors associated with the level of perceived satisfaction in patients treated in 36 nonprofit health clinics that offer comprehensive health care services in four counties in the state of California, United States.

**Methods:**

Cross-sectional analytical study in 14 clinics in four California counties. It consisted of the application of a 30-item questionnaire to determine the degree of patient satisfaction with the clinic. The factorial composition of the quality of care and clinic quality components was analyzed and two factors with an Eigen value greater than 1 were obtained.

**Results:**

A total of 846 responses were registered. Factor analysis identified two underlying dimensions: Physician Attitude and Empathy. It was found that the discordance in language between the physician and the patient generates a difference in the perception of satisfaction. Patients who prefer to speak English have better satisfaction than those who speak Spanish. Spanish speakers who do not have interpreter have lower satisfaction than those who do (*p* < 0,01).

**Conclusions:**

The most important sociodemographic cofactor was language. Satisfaction decreased in Spanish-speaking patients who were not proficient in the use of English since they expressed fewer comments and doubts.

**Supplementary Information:**

The online version contains supplementary material available at 10.1186/s12913-023-09176-2.

## Background

There is a close relationship between quality of service and patient satisfaction. Conceptually, they are two different processes; quality of service includes the evaluation of various factors: doctor-patient relationship; availability; service timeliness; cost/benefit ratio; temporality; user satisfaction, among others. It is considered that there is a reciprocal causal relationship between quality and satisfaction that depends on the time at which it is assessed [1–3].

Healthcare has become an increasingly competitive marketplace, the study of patient satisfaction and the constant measurement of quality of care have contributed to a considerable increase in the quality of services [4].

According to Schiffman and Lazar, in order to understand the quality of the overall service, it is necessary to evaluate users perception, since they determine the extent to which their expectations have been met [5]. For Zeithaml, quality, from the patients’ perspective, can be defined as the difference between their expectations and their perceptions after receiving the requested service [6]. Furthermore, Lloréns and Fuentes consider that knowledge of users’ expectations is the first step in the planning, organization, and provision of a quality service [7]. Aguirre Gas defines quality of care as “[...] providing medical care to the patient, with timeliness, professional competence, safety, and respect for the ethical principles of medical practice, allowing them to meet their health needs and expectations” [8].

It is also considered that the level of satisfaction is the product of two psychological processes, a cognitive one, which includes expectations and perceptions, and an emotional one resulting from the congruence between the user’s expectation and perception. In addition, the subjective evaluation is influenced by past experiences with other health services [9–12].

The characteristics of both the patient and the physician are factors that influence the degree of satisfaction of both. Perception of satisfaction may vary according to age, gender, educational, and socioeconomic level (women and middle- and high-income individuals tend to demand higher quality of medical care than low-income individuals). Physician experience, communication, and trust are the most significant factors in overall patient satisfaction [13, 14]. In addition, older patients’ expectations tend to be higher in terms of communication with their physician, both in terms of education about their disease and explanation of their treatment [10].

The doctor-patient relationship seems to be the most important element in achieving patient and user satisfaction [13]; the patient’s communication with the physician is required in order to express his or her doubts and clarify them determines the level of satisfaction and adherence to treatment. Several authors have also reported that a relaxed atmosphere, the physician’s interest during the consultation, friendliness, a warm tone of voice, empathy, time dedicated to the consultation and privacy are factors that patients consider important when evaluating the service they received [15–18]. Similarly, it has been documented that language differences can significantly affect patients’ perception of the quality of care and reduce levels of satisfaction and adherence to treatment, subsequent visits to the physician [19].

The congruence between physician and patient characteristics has also been studied, that is, to what extent the patient empathizes with the physician. Ross and coworkers found a high level of satisfaction when physicians shared the patient’s place of residence or nationality [20]. Reciprocity between provider and user behaviors has also been related [10].

This document presents the baseline evaluation of level of satisfaction of patients treated in health clinics in the counties of Monterey, Hollister, Tulare and Ventura, in the State of California, United States, participating in Law AB 1045/ Chapter 1157/ The Doctor and Dentists of Mexico Pilot Program (CMPP) [21], the program is aimed at providing care to the immigrant population of that State, who do not speak English and have difficulty accessing health services due to difficulties in understanding their culture. This measurement was made prior to the incorporation of Mexican doctors as providers in these institutions, in the specialties of Family Medicine, Internal Medicine, Obstetrics and Gynecology, and Pediatrics. Subsequently, two more annual measurements will be carried out (2022 and 2023), to compare the level of satisfaction of the patients cared for by bilingual Mexican and English-speaking North American doctors in the same population of the clinics. As it is a pilot law, the results of the evaluation will allow the California Legislature to define its relevance and continuity.

## Method

### Objective

To identify factors associated with the level of perceived satisfaction in patients seen at 36 nonprofit health clinics that provide comprehensive health care services in four counties in the state of California, United States.

An analytical cross-sectional study was conducted by surveying patients or their relatives in 14 medical units participating in CMPP, from Monterey, Hollister, Tulare and Ventura counties in California, United States, during July to September 2021. For confidentiality purposes, we will arbitrarily call the clinics in these four counties: 1,2,3,4 in no specific order.

#### Instrument

A questionnaire was developed with the aim of determining the degree of satisfaction of the patients during their visit based on previous surveys of satisfaction standardized and carried out by the Mexican Institute of Social Security (IMSS), and a primary care center of the Ministry of Health of Mexico (SSA). This questionnaire consisted of 30 multiple-choice questions distributed in two components: sociodemographic characteristics, 6 items and clinical care satisfaction, 24 items on a 4-point Likert-Scale. A pilot test was conducted on twenty volunteers from four health clinics in California and no problems related to wording and semantics were found for the English and Spanish language versions. Questionary script in both languages is included as an appendix.

Fourteen Clinics were selected to represent the four Community Health Clinics, 4 of which will be controls since they will not receive Mexican Physicians and 10 of which will be interventions for the MCPP.

Information was captured using the Offline Surveys application for the Lime Survey version 5.1.0, stored in the SQL server, and analyzed in the Stata 16 software. Satisfaction items were scored with − 1 if the patient was dissatisfied, 0 if they had a neutral perception or did not respond, and 1 if the patient was satisfied. For descriptive analysis, data, tables, and graphics were produced.

#### Participants

All patients seen in the 12 consecutive days that the examiners visited the clinic were invited to voluntarily participate in the survey, either by the evaluators themselves or by clinic staff once they left the medical office after receiving clinical care. The patients who had medical consultation by telephone were also invited to participate in the research at the end of their care by the staff responsible for coordinating the medical consultation. Those who agreed to participate signed an informed consent or verbally accepted (in the case of telephone surveys), approved by the Ethics and Research Committee of the Faculty of Medicine of the National Autonomous University of Mexico (UNAM), number FM/DI/054/2019. Patients from all ages were included. In the case of minors (age up to 17 years and 11 months) and persons who could not respond by themselves because of a disability, survey was applied to their parents or guardians to respond on behalf of the patient.

Before the survey application, the examinator asked the participant for his preferred language and the survey’s version was presented, according to their choice (Spanish or English). All participants were surveyed by a bilingual physician unrelated to clinic.

Before the survey application, the examinator asked the participant for his preferred language and the survey’s version was presented, according to their choice (Spanish or English). All participants were surveyed in private.

#### Statistical analysis

Factorial composition of doctor’s attitude and physician empathy perception components were analyzed and two factors with an eigen value above 1 were obtained. The relevance of the technique was examined through two procedures:

- the examination of the Kaiser-Mayer-Olkin sample adequacy measure obtaining a value of 0.842, and
- the Bartlett sphericity test to verify the interdependence of the items with a significative test (*p* < 0.05)

Factor extraction was performed using the principal axis method and Cronbach’s Alpha Index was calculated for the resulting factor after the factor rotation using the orthogonal method. Resulting factors were named after the items that had more weight. (Omit on factor.)

By author’s consensus, satisfaction/insatisfaction cutpoint was established in 0, as neutral responses were punctuated 0, favorable responses were punctuated positively, and not favorable responses were punctuated negatively. Additionally, participants with factor value higher than + 0.5 factor were considered clearly satisfied and those with value lower than − 0.5 were considered clearly unsatisfied.

Physician-patient linguistic discordance, patient’s age, sex, scholarity, ethnical group, marital status occupation and doctor’s specialty were evaluated with Kruskall-Wallis test to determine whether any resulting factor is dependent on any of them. Categories with less than 5 observations were grouped in a special category.

## Results

**Fig. 1:**
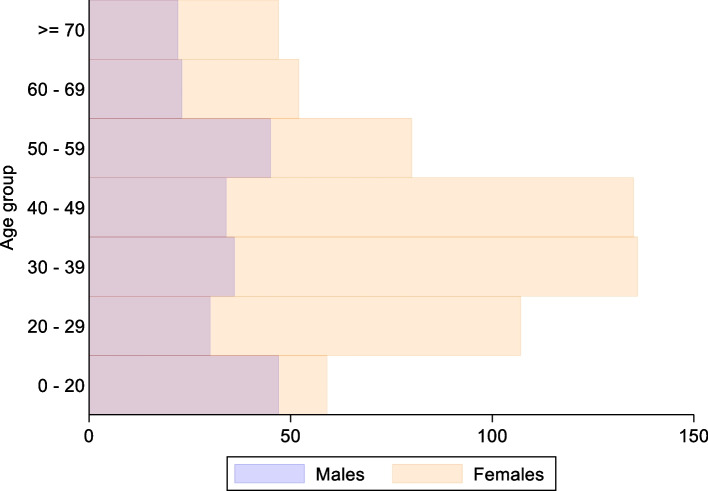
Age and sex distribution of participants

Eight hundred seventy-six patients agreed to participate and eight hundred forty-six fully responded surveys were included: 232 from male patients and 603 from female patients. 11 patients prefer not to specify sex. The age ranges with the largest number of participants are the groups of 30 to 39 and 40 to 49 years (20% each). See Fig. 1.

According to County, 54% of the sample participants are from Tulare, 23% from Hollister, 14% from Ventura, and 9% from Monterey. 71% of those served are Latino, 11% Caucasian, 1% African American, and the remainder from other demographic groups.

Most people receiving care at the clinics are homemakers (25%), farmworkers (18%), and service workers (12%).

Most of the people that received medical care have High School as their maximum degree of studies (35.1%); 15.6% have studies up to middle school, and 14.2% have completed elementary school.

Most of the patients (70%), who participated were seen by a family doctor. 92% of the patients claimed to have been treated in their clinic before. 43% commented that the doctor spoke to them in Spanish during the consultation and 42% required an interpreter.

### Factorial analysis

The factor analysis showed two factors that met the criteria and are presented in Table 1.

**Table 1:** Factors load and consistency

Item	Question	Factor load	Consistency (Cronbach’s α)
**Doctor’s attitude**			**0.8218**
8	1. What did you think of the doctor’s explanation of your condition?	0.4131	0.7936
11	2. How did you feel the doctor treated you?	0.6821	0.7565
12	3. How would you rate the care you received from the doctor at the clinic?	0.7120	0.7723
17	4. Would you like to be treated by the same physician who treated you?	0.5829	0.7796
**Physician empathy perception**			**0.7563**
4	5. Did the physician listen to your explanation of your condition?	0.6080	0.8000
5	6. Do you feel that the physician understood your symptoms or complaints that prompted the consultation?	0.6668	0.6689
7	7. Did the doctor answer all your questions?	0.6177	0.6437
9	8. Did the physician show interest in resolving your condition?	0.5704	0.7030

Table 2 contains the proportion of participation by item and factor mean with 95% IC.

**Table 2:** Factorial means by sociodemographic factors of the sample

Characteristic	Proportion %	Factor 1. Doctor’s attitude	Factor 2. Physician empathy Mean [CI 95%]
**Overall**	100	0.00 [− 0.05 – 0.05]	0.05 [− 0.01 – 0.12]
**Sex**			
Man	27.0	0.07 [0.01 – 0.14]	- 0.02 [− 0.13 – 0.08]
Woman	70.3	- 0.03 [− 0.10 – 0.04]	0.01 [− 0.05 – 0.07]
No response	0.1		
**Age Group**			
0 – 20	12.6	0.06 [− 0.01 – 0.14]	0.18 [0.07 – 0.28] *
20 – 29	16.3	0.09 [0.01 – 0.17] *	0.01 [− 0.13 – 0.15]
30 – 39	20.4	0.02 [− 0.10 – 0.16]	- 0.01 [− 0.13 – 0.11]
40 – 49	20.3	0.01 [− 0.09 – 0.13]	- 0.03 [− 0.16 – 0.08]
50 – 59	15.1	- 0.16 [− 0.38 – 0.06]	0.01 [− 0.11 – 0.14]
60 – 69	9.1	0.04 [− 0.05 – 0.15]	0.16 [0.06 – 0.26] *
70 and over	6.0	- 0.10 [− 0.34 – 0.14]	- 0.49 [− 0.87 – - 0.11] *
**County**			
1	8.7	0.13 [0.03 – 0.23] *	0.04 [− 0.24 – 0.14]
2	13.2	0.04 [− 0.09 – 0.17]	0.21 [− 0.14 – 0.28]
3	23.1	- 0.20 [− 0.37 – 0.03]	0.00 [− 0.11 – 0.11]
4	51.8	0.05 [− 0.01 – 0.12]	- 0.05 [− 0.14 – 0.02]
**Physician’s specialist**			
Family physician	70.0	- 0.02 [− 0.10 – 0.05]	- 0.07 [− 0.10 – 0.05]
Internist	2.18	- 0.37 [− 0.94 – 0.19]	0.13 [− 0.21 – 0.48]
Gynecologist	5.51	0.20 [− 0.14 – 0.27] *	- 0.04 [− 0.34 – 0.25]
Pediatrician	14.8	0.08 [− 0.03 – 0.20]	0.12 [0.03 – 0.22] *
Other	7.4	0.13 [− 0.04 – 0.22]	0.22 [0.11 – 0.32]
**Population group**			
Latin	71.6	- 0.01 [− 0.07 – 0.05]	0.01 [− 0.04 – 0.07]
Caucasian	11.0	- 0.04 [− 0.24 – 0.16]	- 0.11 [− 0.32 – 0.08]
Afro-Americans	1.6	- 0.13 [− 0.72 – 0.45]	0.05 [− 0.30 – 0.41]
Other	12.6	0.10 [− 0.01 – 0.21]	- 0.01 [− 0.19 – 0.16]
No answer / did not respond	3.0	- 0.10 [− 0.43 – 0.22]	0.23 [− 0.01 – 0.47]
**Maximum degree of studies**			
None	6.5	- 0.12 [− 0.41 – 0.16]	0.02 [− 0.14 – 0.18]
Elementary school (unfinished)	13.4	- 0.12 [− 0.31 – 0.06]	- 0.07 [− 0.22 – 0.07]
Elementary school	14.2	- 0.06 [− 0.25 – 0.12]	- 0.05 [− 0.21 – 0.10]
Middle school	15.6	0.11 [0.04 – 0.18] *	0.06 [− 0.06 – 0.17]
High school	35.1	- 0.01 [− 0.10 – 0.08]	- 0.02 [− 0.13 – 0.07]
University	10.6	0.14 [0.07 – 0.20] *	0.12 [− 0.02 – 0.26]
No answer / did not respond	4.3	0.15 [0.05 – 0.24] *	0.18 [0.02 – 0.33] *
**Occupation**			
Housewife	23.7	0.03 [− 0.07 – 0.14]	- 0.05 [− 0.16 – 0.05]
Self-employed worker	3.4	0.20 [0.15 – 0.26]	- 0.10 [− 0.42 – 0.20]
Student	7.8	0.10 [0.01 – 0.18]	0.07 [− 0.09 – 0.24]
Professional	10.2	0.04 [− 0.07 – 0.15]	- 0.03 [− 0.23 – 0.16]
Unemployed	9.4	0.04 [− 0.09 – 0.18]	0.21 [0.08 – 0.33]
Pensioner or retiree	6.2	- 0.32 [− 0.71 – 0.05]	- 0.09 [− 0.35 – 0.17]
Agricultural worker / Domestic worker	21.6	−0.10 [− 0.25 – 0.04]	0.02 [− 0.09 – 0.14]
Service / industrial worker	17.2	0.06 [− 0.04 – 0.17]	- 0.03 [− 0.18 – 0.11]
**Civil status**			
Single	36.7	0.05 [− 0.01 – 0.11]	0.03 [− 0.05 – 0.12]
Married / Free union	50.5	- 0.02 [− 0.11 – 0.06]	- 0.02 [− 0.09 – 0.05]
Divorced / Separated	6.7	0.05 [− 0.09 – 0.19]	0.12 [− 0.02 – 0.27]
Widower / Widow	3.3	- 0.26 [− 0.61 – 0.20]	- 0.42 [− 0.91 – 0.06]
No answer / did not respond	2.6	- 0.19 [− 0.81 – 0.41]	0.17 [− 0.15 – 0.51]
**Who is the patient living with?**			
Alone	6.8	- 0.02 [− 0.21 – 0.16]	0.12 [− 0.03 – 0.26]
With partner	14.5	- 0.07 [− 0.23 – 0.08]	- 0.03 [− 0.18 – 0.12]
With children	13.2	- 0.08 [− 0.28 – 0.12]	0.02 [− 0.13 – 0.17]
With couple and sons	35.7	- 0.01 [− 0.10 – 0.10]	- 0.01 [− 0.09 – 0.08]
With other relatives	20.4	0.09 [− 0.04 – 0.15]	- 0.07 [− 0.21 – 0.08]
With friends	6.7	0.03 [− 0.11 – 0.17]	0.13 [− 0.00 – 0.26]
With strangers	0.3	0.27 [0.08 – 0.46] *	- 0.35 [− 0.81 – 0.11]
No answer / did not respond	1.9	0.21 [0.05 – 0.37] *	0.15 [− 0.15 – 0.47]
**Previously attended on clinic?**			
Yes	92.5	0.00 [− 0.06 – 0.05]	0.00 [− 0.05 – 0.05]
No	4.2	0.06 [− 0.20 – 0.32]	- 0.03 [− 0.25 – 0.19]
No answer	3.2	0.05 [− 0.31 – 0.42]	−0.01 [− 0.40 – 0.38]
**Preferred Language**			
Spanish	66.2	- 0.02 [− 0.10 – 0.05]	−0.11 [− 0.18 – - 0.03] *
English	33.7	0.04 [− 0.01 – 0.11]	0.21 [0.15 – 0.27] *
**Physicians spoke in Spanish?**			
Yes	43.7	0.04 [− 0.03 – 0.12]	0.00 [−0.09 – 0.08]
No	51.2	- 0.04 [− 0.12 – 0.04]	0.01 [− 0.06 – 0.08]
No answer	5.0	0.02 [− 0.20 – 0.24]	0.11 [− 0.52 – 0.28]
**Patient needed a translator/interpreter during consultation?**			
Yes	42.7	- 0.04 [− 0.14 – 0.04]	0.02 [− 0.05 – 0.10]
No	57.3	0.02 [− 0.05 – 0.09]	- 0.01 [− 0.09 – 0.06]

### Doctor’s attitude

**Fig. 2:**
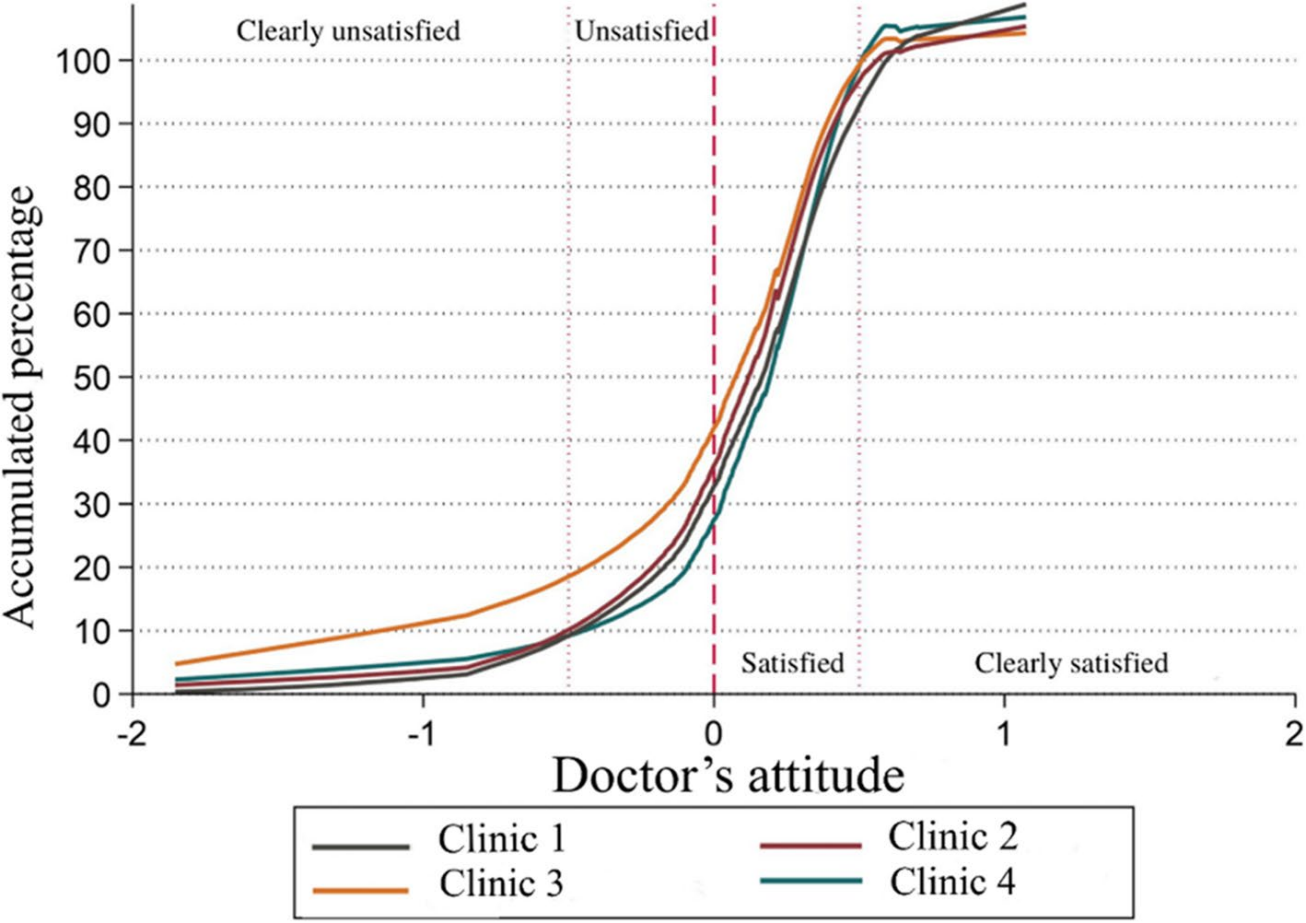
Factor 1 comparison among counties (rural clinics)

Of the participants, 83% had a positive value on this factor considering them as satisfied and 1.94% scored above 0.5 in this factor, considering them as clearly satisfied. On the other hand, 10.6% were partially unsatisfied and 7.38% were fully unsatisfied (using a cut point in - 0.5). County “3” had the lowest overall satisfaction even when they have 73.7% patients satisfied. People between 20 and 29 years showed a better satisfaction than other groups. See Fig. 2.

It was found that the language of attention generates a difference in the perception of this satisfaction component. Those patients who prefer English and do not require a translator have better satisfaction than the rest, compared to those who prefer Spanish and use a translator having the worst satisfaction in this component (*p* < 0.05).

The demographic group to which they belong is also associated with changes in satisfaction, with the Latino group having less satisfaction than the Caucasian and African American groups. Those who did not specify a demographic group also have a better perception.

Occupation also generates differences in this factor, self-employed workers and students have a good perception of clinical care. The group with the worst perception is pensioners/retired and agricultural workers (*p* < 0.05).

There were no significant differences in this factor by education (*p* = 0.16), marital status (*p* = 0.25), with whom the patients live (*p* = 0.11) specialist type (*p* = 0.12).

### Physician empathy perception

**Fig. 3:**
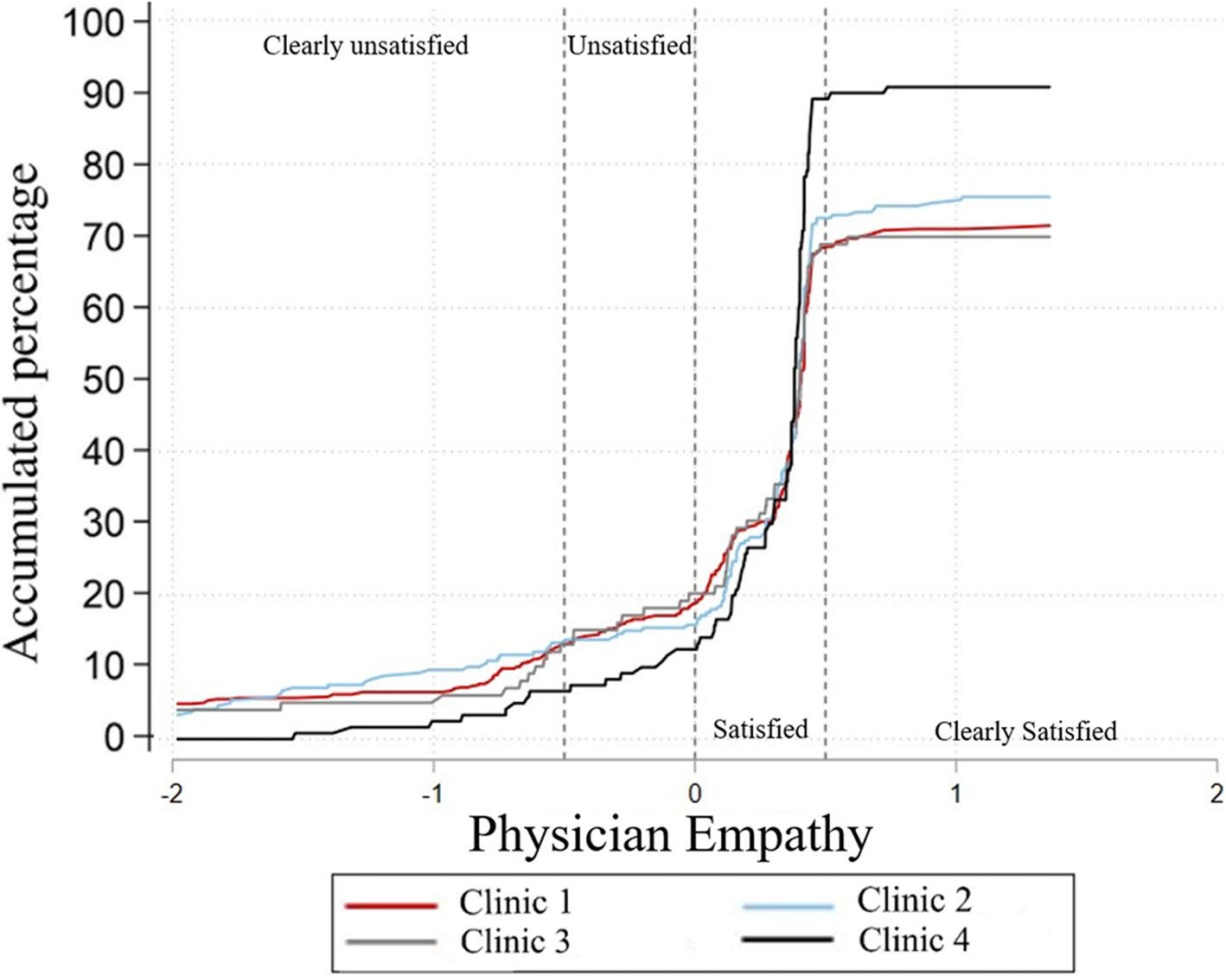
Factor 2. Physician empathy perception by county (rural clinic)

Seventy-three percent of the participants were partially satisfied with the doctor’s empathy and 3.7% totally satisfied. Of the 23.3% of patients who said they were dissatisfied, 16.5% said they were totally dissatisfied. According to the county of care, no difference was found between them in terms of physician empathy. Patients in the age groups 0-19 years and 60-69 years showed better satisfaction in this component, and patients who were 70 years and older had a lower level of satisfaction. See Fig. 3.

It was found that the language of attention generates a difference in the perception of satisfaction. Those patients who prefer English, regardless of the use of the family interpreter or health worker, have better satisfaction than those who speak Spanish. Spanish speakers who do not have interpreter support have lower satisfaction than those who do have interpreter support (*p* < 0.01).

The demographic group is not associated with this component (*p* = 0.35). According to the occupation, there is a difference in satisfaction with the doctor-patient relationship. The group of unemployed and students have a better satisfaction than the rest. The group of self-employed workers and pensioners are the ones with the worst satisfaction of the group.

**Fig. 4:**
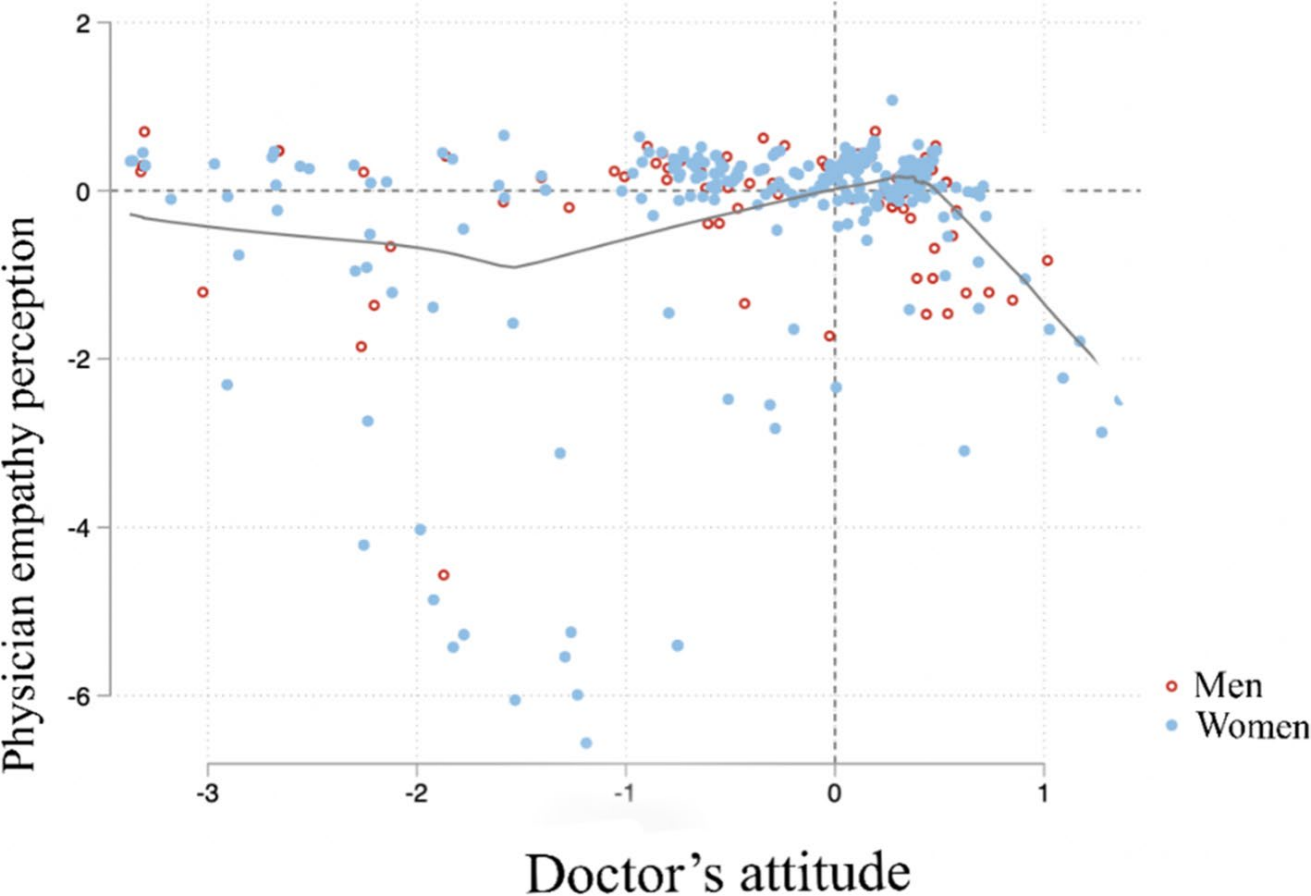
Correlation between factors

There were no significant differences in the opinion of this component by schooling (*p* = 0.41). There were no significant differences in the opinion of this component regarding the specialty of the physician (*p* = 0.22). The factors were partially correlated (slope = 0.2138) as shown in Fig. 4. Most values are concentrated in the upper right quadrant, with 65% percent of the population.

## Discussion

This research obtained evidence of the satisfaction levels of the patients who received care in the clinics that participated in the California-Mexico Pilot Program. The overall satisfaction index regarding the physician’s attitude was found to be 83%, with a dissatisfaction index of 17.8%, of which 7.3% of the patients were clearly dissatisfied. As for the physician’s empathy (as part of a good doctor-patient relationship), 77% of the participants were found to be satisfied.

The most important sociodemographic cofactor in terms of correlation weight was language for both factors. Satisfaction levels and language mismatch (physicians and patients have discordance in the language they often speak) are positively associated. This is reflected in the fact that patients who preferred to respond in Spanish and use an interpreter were notably more dissatisfied than those who responded in English and did not use an interpreter [22]. This phenomenon has already been reported by other authors in various studies, referring to the importance of the language barrier, generating less possibility of expressing themselves to the physician and resolving their doubts [23]. This population expresses fewer concerns and questions compared to patients who are fluent in English (up to three times less), so they may have lower health care outcomes, decreased adherence to treatment and fewer visits to the doctor [22, 24–29]. This could correspond to farm and domestic workers, who in many cases, due to their low level of education, do not communicate adequately with their physicians because they do not speak English, which results in lower satisfaction with health services [29, 30].

Another interesting aspect was that the use of interpreters reduces the degree of dissatisfaction of patients with respect to empathy with their physician [31], which corresponds to the reports by Wilson, et al. and Lee, et al., in relation to language barriers, who state that translation by untrained family members can sometimes generate a greater degree of dissatisfaction, acknowledging that specialized interpreters can provide a greater benefit, similar to that achieved when physician and patient use the same language. The use of interpreters may also increase the possibility of attracting more patients who may not seek medical care because of the language barrier [23, 24].

Other sociodemographic cofactors that were associated with the level of satisfaction, were age, ethnicity, and occupation. The main relationships between these cofactors are: 1) Patients from 0 to 20 years of age had greater satisfaction with the doctor’s empathy than other ages, which is clearly related to having a greater command of English, being mostly children of first-generation immigrants, born in California. Likewise, in this study it is associated with the highest level of satisfaction reported in pediatric care. Selecting English as preferred language was more common in people with lower age. Patients over 70 years of age, mostly pensioners or retirees had a low level of satisfaction in both factors, which has been reported in other papers [12, 31], where it is mentioned that the perception of satisfaction may be different according to age, sex, educational and socioeconomic level, particularly women, and people with medium and high economic income, who tend to be more demanding with the quality of medical care they receive [12, 31].

In the case of elderly retirees, this can be explained by the type of insurance they had previously contracted; they assume that paying taxes gives them the right to demand higher quality services, as expressed verbally, with relative frequency. Another documented aspect is that older people require more clarity and time in their communication with the physician to understand their illness and the treatment administered [15].

County differences were adjusted in other cofactors and the remaining variation is significant for factor 1 but not for factor 2. A better experience was observed in clinic 1 for factor 1 and 2 for factor 2 (non-significant). These results lead to explore other possible cofactors as physician’s training or experience.

Regarding the limitations of the study, it is important to mention that it was developed in clinics in four California counties, whose predominant population is of Mexican origin, which does not correspond to other regions of the United States. Likewise, it should be considered that although the response rate of those invited to participate was very high, the exact refusal rate is not known because some of the patients were invited by telephone by clinic personnel after completing their telephone medical consultation and the researchers did not have access to this information, however, it is estimated that it was approximately 10%.

## Conclusions

This is the first study conducted to determine the satisfaction levels of the health clinics participating in the AB 1045/Chapter 1157/ The Doctor and Dentists from Mexico Pilot Program, prior to the incorporation of 30 Mexican physicians (baseline assessment). Two more measurements of patient satisfaction will be made after the Mexican doctors start working in the clinics (end of 2022 and 2023), comparing the satisfaction of the patients they have treated and contrasting the results with the ratings of patients treated by North American doctors working in the same clinics serving immigrant population. The health clinics participating in this research have a high degree of satisfaction for the factors of physician attitude and empathy. However, there are 17 and 23% of patients, respectively, who could improve their satisfaction if the reported findings are considered.

It was found that the most important sociodemographic cofactor in determining patient satisfaction was language for both factors. Satisfaction levels decreased when physicians and patients had language discordance (English-Spanish), and even the use of non-professional interpreters did not completely reverse patient dissatisfaction [32]. It is important to emphasize that in the case of California, most of the low socioeconomic population is of Latino origin, immigrants, who in many cases do not speak English and are first generation farm workers. There are several factors associated with the decreased patient satisfaction in clinics, which with appropriate interventions, such as adequate patient-physician communication, could improve the patient’s experience in health clinics.

The CMPP program is expected to address and resolve language discordance in California clinics. And increase the efficiency and accessibility of health services to improve the quality of life of the Latino population in the United States.

## Supplementary Information

**Additional file 1.**

## Data Availability

The data that support the findings of this study are available from Miguel Ángel Fernández-Ortega but restrictions apply to the availability of these data, which were used under license for the current study, and so are not publicly available. Data are however available from the authors upon reasonable request and with permission of the participating clinics.
