# Supplementary material for: Patient dissatisfaction associated with physician-patient linguistic discordance in California clinics: an analytical cross-sectional study
Source: BMC Health Serv Res. 2023 Feb 23;23:189. doi: 10.1186/s12913-023-09176-2 (PMC9948301; doi:10.1186/s12913-023-09176-2)
Supplement: Supplementary file 1 — Additional file 1. [file 12913_2023_9176_MOESM1_ESM.docx]

**PATIENT SATISFACTION SURVEY**

**Bilateral Program for Access to health services provided by Mexican doctors within migrant communities in California**

To help us improve our patient care, you are invited to participate anonymously in this survey measuring patient satisfaction with the medical services offered at this Health Clinic. Your responses are confidential and will not affect the care that you receive or will receive in the future.

If you would like to collaborate, please answer the following questions with the most accurate response you can give. If a question is about something that you haven’t experienced, please answer **‘Does not apply.’**

Thank you for participating.

**Survey number Medical Unit Code (01) (02) (03) (04) Date**

| **No.** | **Question** | **Answers** |
| --- | --- | --- |
| **1** | Have you previously received medical care in this clinic? | 1. Yes 2. No 3. Does not apply 4. Other _____ |
| **2** | Did the doctor who saw you on your visit speak to you in Spanish? | 1. Yes 2. No 3. Don’t know or does not apply 4. Other______ |
| **3** | Have you or a family member needed a  translator during your doctor’s appointment? | 1. Yes 2. No 3. Don’t know or Does not apply 4. Other |
| **4** | What type of specialist did you see? | - 1. General practitioner (family doctor)   2. Internal medicine specialist   3. Gynecologist   4. Pediatrician   5. Don’t know or Does not apply   6. Other |
| **5** | How old are you (in years)? | 1. Younger than 20 2. 20 - 29 3. 30 - 39 4. 40 - 49 5. 50 - 59 6. Older than 60 |
| **6** | Gender | 1. Male 2. Female 3. Prefer not to say 4. Other |
| **7** | Do you identify yourself with one of the following ethnic groups? | 1. Latino 2. White / Caucasian 3. Black / African American 4. Asian 5. Indian (from India) 6. Native American / Alaskan native 7. Hawaiian / Pacific Islander 8. Unknown 9. Other |
| **8** | Marital status | - 1. Single / unmarried   2. Married / Cohabiting   3. Divorced / separated   4. Widow / widower   5. Does not apply   6. Otro ______ |
| **9** | Who do you live with? | 1. I live alone 2. With my spouse or partner 3. With my children 4. With my spouse / partner and children 5. With other family members 6. With friends 7. With work colleagues 8. With people I don’t know 9. Other |
| **10** | Educational level | 1. None 2. Incomplete elementary school 3. Completed elementary school 4. Incomplete Middle school 5. Complete Middle School 6. Incomplete High school 7. Complete High School 8. University or higher 9. Don’t know or does not apply 10. Other |
| **11** | Primary occupation | 1. Agricultural laborer 2. Service worker (waiter/waitress, electrician, fire fighter, police officer, etc.) 3. Construction worker 4. Industrial worker 5. Self-employed 6. Domestic worker 7. Homemaker 8. Student 9. Professional 10. Unemployed 11. Retired or pensionate 12. Does not apply 13. Other |
| **12** | The last three times you have come to this clinic, what kind of service did you have?  **MARK ALL THAT APPLY** | - 1. In-person visit   2. Over the internet   3. On the telephone |
| **13** | How do you prefer to be attended by the doctor? | 1. In person 2. Over the internet 03 On the telephone 3. Does not apply 4. Other |
| **14** | During your visit, did you have the chance to ask the doctor all your questions and concerns about your health? | - 1. Yes, in detail   2. Yes, partially   3. I didn’t want to  1. I didn’t have the chance to 2. Don’t know 3. Does not apply 4. Other |
| **15** | Did the doctor take sufficient time to explain to you about your illness, or about the reason for your visit? | 1. Very suitable 2. Enough 3. Not enough 4. Very insufficient 5. Don’t know or Does not apply 6. Other_____ |
| **16** | How much did the doctor ask for your opinion during the visit? Did she/he take it into consideration? | 1. Too much 2. Much 3. A little bit 4. Not at all 5. Don’t know or does not apply 6. Other |
| **17** | During this medical appointment did the doctor listen carefully about your illness? | 1. Yes 2. No 3. Don’t know o Does not apply 4. Other |
| **18** | Do you feel that the doctor understood your symptoms or problems that were the reason for your visit? | 1. Completely 2. Partially 3. A little bit 4. No 5. Don’t know / Does not apply 6. Other _____ |
| **19** | Do you feel that the examination that the doctor gave you was complete? | - 1. Complete   2. Incomplete   3. He/she didn’t examine   4. Don’t know / Does not apply   5. Other _________ |
| **20** | Did the doctor answer all your questions? | 1. Yes 2. No 3. Don’t know / Does not apply 4. Other ______ |
| **21** | How was the explanation that the doctor gave you regarding the reason for your visit? | 1. Very clear 2. Clear 3. Confusing 4. Very confusing 5. Don’t know / Does not apply 6. Other______ |
| **22** | Did the doctor show interest in resolving your problem? | - 1. Yes  1. No 2. Don’t know / Does not apply 3. Other _____ |
| **23** | Do you think that, in order to improve the quality of your visit, the doctor took into consideration your language, religion, beliefs, and place of birth? | 1. Yes 2. Somewhat 3. No 4. Don’t know / Does not apply 5. Other _____ |
| **24** | How was your doctor’s attitude with you? | - 1. Friendly / Warm   2. Indifferent   3. Rude /Aggressive /Cold   4. Don’t know / Does not apply   5. Other ________ |
| **25** | How would you rate the care that **the doctor**  gave you in the clinic? | 1. Very good 2. Good 3. Bad 4. Very bad 5. Don’t know / Does not apply 6. Other______ |
| **26** | How would you rate the care that **the staff** gave you in the clinic? | 1. Very good 2. Good 3. Bad 4. Very bad 5. Don’t know / Does not apply 6. Other _______ |
| **27** | In the last six months, have you noticed any improvements in the quality of service at the Clinic? | Yes, it has improved  02 It has stayed the same 03 It has worsened  04 Don’t know / Does not apply   1. Other _______ |
| **28** | If a friend or family member needed medical care, would you recommend that they come to this clinic? | 1. Yes 2. No 3. Don’t know / Does not apply 4. Other |
| **29** | In general, did you receive the medical care that you hoped for? | 1. Yes 2. No 3. Don’t know / Does not apply 4. Other |
| **30** | Would you want the doctor who attended you to attend you again? | 1. Yes 2. No 3. Not sure or does not apply 4. Other ______ |
